# Laser Scanning Confocal Microscopy Was Used to Validate the Presence of *Burkholderia pseudomallei* or *B. mallei* in Formalin-Fixed Paraffin Embedded Tissues

**DOI:** 10.3390/tropicalmed5020065

**Published:** 2020-04-29

**Authors:** Kei Amemiya, Xiankun Zeng, Jeremy J. Bearss, Christopher K. Cote, Carl Soffler, Robert C. Bernhards, Jennifer L. Dankmeyer, Wilson J. Ribot, Sylvia R. Trevino, Susan L. Welkos, Patricia L. Worsham, David M. Waag

**Affiliations:** 1 Bacteriology Division, United States Army Medical Research Institute of Infectious Diseases, Fort Detrick, MD 21702, USA; Christopher.k.cote.civ@mail.mil (C.K.C.); Carl.soffler.mil@mail.mil (C.S.); Jennifer.l.dankmeyer.ctr@mail.mil (J.L.D.); Wilson.J.ribot.civ@mail.mil (W.J.R.); sylivia.r.trevino.civ@mail.mil (S.R.T.); Susan.l.welkos.vol@mail.mil (S.L.W.); Patricia.l.worsham.civ@mail.mil (P.L.W.); Dmwaag@comcast.net (D.M.W.); 2 Pathology Division, United States Army Medical Research Institute of Infectious Diseases, Fort Detrick, MD 21702, USA; Xiankun.zeng.ctr@mail.mil (X.Z.); Jeremy.j.bearss.mil@mail.mil (J.J.B.); 3 U.S. Army Combat Capabilities Development Command Chemical Biological Center, 8198 Blackhawk Road, Aberdeen Proving Ground, MD 21010, USA; Robert.c.bernhards.civ@mail.mil

**Keywords:** *Burkholderia pseudomallei*, melioidosis, *Burkholderia mallei*, glanders, laser scanning confocal microscopy, formalin-fixed paraffin embedded tissue, animal models, microorganism

## Abstract

*Burkholderia pseudomallei* and *B. mallei* are Gram-negative, facultative intracellular bacteria that cause melioidosis and glanders, respectively. Currently, there are no vaccines for these two diseases. Animal models have been developed to evaluate vaccines and therapeutics. Tissues from infected animals, however, must be fixed in formalin and embedded in paraffin (FFPE) before analysis. A brownish staining material in infected tissues that represents the exopolysaccharide of the pathogen was seen by bright field microscopy but not the actual microorganism. Because of these results, FFPE tissue was examined by laser scanning confocal microscopy (LSCM) in an attempt to see the microorganism. Archival FFPE tissues were examined from ten mice, and five nonhuman primates after exposure to *B. pseudomallei* or *B.* *mallei* by LSCM. Additionally, a historical spleen biopsy from a human suspected of exposure to *B. mallei* was examined. *B. pseudomallei* was seen in many of the infected tissues from mice. Four out of five nonhuman primates were positive for the pathogen. In the human sample, *B. mallei* was seen in pyogranulomas in the spleen biopsy. Thus, the presence of the pathogen was validated by LSCM in murine, nonhuman primate, and human FFPE tissues.

## 1. Introduction

Melioidosis is caused by the Gram-negative, facultative intracellular pathogen *Burkholderia pseudomallei*. It is endemic in Southeast Asia and northern Australia and appears to be much more widely distributed than originally reported [1]. It can be found in soil, wet lands, and water, and the incidence of melioidosis appears to increase during the raining season [2]. Exposure to *B. pseudomallei* can be through cutaneous inoculation, ingestion of contaminated water or food, or by inhalation. Infection by *B. pseudomallei* is the third leading cause of disease in Southeast Asia behind tuberculosis and AIDs, and pneumonia is the most common clinical presentation of melioidosis. Host risk factors, such as diabetes, excess alcohol consumption, and renal and lung disease, significantly influence the susceptibility to infection [3,4]. Treatment of melioidosis is difficult because of similar clinical presentations of other infections, such as tuberculosis, and the intrinsic resistant to common antibiotics by the organism [5]. At present, there is no efficacious vaccine against melioidosis. Because of its potential use as a biological agent, the Centers for Disease Control and Prevention (CDC) considers *B. pseudomallei* and its closely related species *B. mallei*, which causes glanders, as Tier 1 biological agents.

Animal models of melioidosis and glanders were developed for the evaluation of therapeutics or vaccines [6,7,8,9]. Formalin-fixed, paraffin-embedded (FFPE) tissue from these studies were examined by immunohistochemical (IHC) analysis with bright field microscopy. In infected tissue, such as spleens (where the pathogens were isolated in unfixed tissue), a brownish positive signal was seen primarily in pyogranulomatous lesions that are associated with *B. pseudomallei* or *B. mallei* infections. This positive signal comes from the recognition of the exopolysaccharide that is common between these two pathogens, but the actual microorganism was not visualized [10,11,12]. There was a question if laser scanning confocal microscopy (LSCM) was used, could the pathogen in FFPE infected tissue be seen. In an initial study, the pathogen was seen in an archival FFPE tissue by LSCM. Thus, a retrospective study of archival tissue from our animal model studies was started with LSCM in order to see if the pathogen could be visualized in other FFPE tissues as a proof of concept. A rabbit polyclonal antibody raised against a formalin-fixed, *B. mallei* whole-cell antigen was used as the primary antibody to analyze FFPE tissue. Furthermore, different antibody preparations were evaluated with LSCM to visualize the pathogen in FFPE infected tissue. In the following report, examples of the presence of *B. pseudomallei* or *B. mallei* in archival tissues from our animal model studies using LSCM were presented. In addition, the possible presence of *B. mallei* in a historical biopsy of a spleen from a human suspected of exposure to *B. mallei* was presented. Finally, different antibody preparations were shown to be used with LSCM to visualize the pathogen in FFPE tissues.

## 2. Materials and Methods

### 2.1. Bacterial Strains, B. pseudomallei K96243 Antibody, and Human Tissue 

*B. mallei* GB18-3 was obtained from the Bacteriology Division culture collection at the U.S. Army Medical Research Institute of Infectious Diseases (USAMRIID), Fort Detrick, Frederick, Maryland, and it had been passed through hamsters 3 times [13]. Single use stock cultures of *B. pseudomallei* K96243 were obtained from the Unified Culture Collection (UCC) at USAMRIID. A rabbit antibody preparation made against an extract of irradiated, whole-*B. pseudomallei* K96243 (IRBpK) cells was a kind gift from Robert Ulrich (USAMRIID). Human tissue from a patient suspected of exposure to *B. mallei* was obtained from the Joint Pathology Center (Silver Spring, MD, USA).

### 2.2. Growth of Bacterial Strains and Antigen Preparation

The following procedure describes the general growth conditions and preparation of a whole-cell, bacterial antigen of *B. mallei* [13] or *B. pseudomallei*. All procedures were performed under biosafety level 3 (BSL3) conditions and culture/cell manipulations were carried out in a biosafety hood. Two hundred milliliters of 4% glycerol tryptone broth (GTB) (Difco, ThermoFisher Scientific, Walthman, MA, USA) in a 1L flask was inoculated with 5 µl of a stock culture of the organism, and the culture incubated with shaking at 200 rpm overnight at 37 °C (16–18 h). After overnight growth, the culture was placed into 50 mL conical tubes (not more than half full), and the tubes were centrifuged for 25 min at 3700 rpm in a swinging bucket rotor at 4 °C. The culture supernatants were discarded and cell pellets were suspended in 1.0 mL of Hanks Balanced Salt Solution (HBSS with calcium and magnesium, ThermoFisher Scientific). The cell suspensions were combined and formaldehyde (ThermoFisher Scientific) was added to a final concentration of 4%. The cells were left in the formalin solution for 24 h at 4 °C, and then the cells were washed twice with cold HBSS. Ten percent of the total volume was used to test for sterility on sheep blood agar plates that were incubated at 37 °C for 3 days. After validation of the sterility of the formalin-fixed cells, the cells were dialyzed for 3 days against 1 L of water with daily changes (Spectra 3000 MW-cut off, ThermoFisher Scientific) at 4 °C. The dialyzed cells were centrifuged and cell pellets were suspended in sterile water. The absorbance of the cells suspension was compared to a standard curve to calculate the protein concentration, and the cells were stored in aliquots at −70 °C. 

### 2.3. Production of Rabbit Polyclonal Antibodies Against B. mallei or B. pseudomallei

Two methods were used to produce rabbit polyclonal antibodies against formalin-treated whole-cells. In the first method, formalin-treated *B. mallei* (fBm) GB18-3 cells were formulated with Ribi TriMix as the adjuvant (Ribi ImmunoChem Research Inc., Hamilton, MT, USA) [13]. In the second method, formalin-treated *B*. *pseudomallei* K96243 (fBpK) cells were formulated with Freund’s complete adjuvant (FCA) or Freund’s incomplete adjuvant (FIA) (Sigma-Alrich, Saint Louis, MO, USA). The general procedure to generate polyclonal antibodies in 2 female NZW rabbits (~2.5 kg) were as follows (Covance Research Products, Denver, PA, USA): prebleed, 21 days before the primary vaccination; primary vaccination, 250 µg of fBpK in FCA; 3 boost (21 days apart) vaccinations starting 21 days after the primary vaccination, 125 µg of fBpK in FIA; terminal bleed, 14 days after the last boost. Antibody (IgG) titers against IRBpK, fBpK, and fBm cells were determined at least twice by ELISA as previously described [14]. See Table A1 in Appendix A for antibody titers of antibodies used in the present study. No new animals were used at USAMRIID for this report.

### 2.4. Immunohistochemistry

Immunohistochemistry (IHC) was performed using the Dako Envision system (Dako Agilent Pathology Solutions, Carpinteria, CA, USA). Briefly, after deparaffinization, peroxidase blocking, and antigen retrieval, sections were covered with a rabbit polyclonal anti-*B. mallei* or *B. pseudomallei* antibody (USAMRIID, Frederick, MD, USA) at a dilution of 1:6000 and incubated at room temperature for forty five minutes. They were rinsed, and the peroxidase-labeled polymer (secondary antibody) was applied for thirty minutes. Slides were rinsed and a brown chromogenic substrate 3,3′ Diaminobenzidine (DAB) solution (Dako Agilent Pathology Solutions) was applied for eight minutes. The substrate–chromogen solution was rinsed off the slides, and the slides were counterstained with hematoxylin and rinsed. The sections were dehydrated, cleared with Xyless, and then coverslipped. Stained sections were digitized and examined with Aperio Image Scope software (Aperio Technologies, Vista, CA). The specimens were examined with an Olympus BX53 microscope (Olympus America, Center Valley, PA, USA). 

### 2.5. Immunofluorescence and Laser Scanning Confocal Microscopy Imaging

Formalin-fixed paraffin embedded (FFPE) tissue sections were deparaffinized using xylene and a series of ethanol washes before staining single sections with H&E. 0.1% Sudan black B (Sigma-Alrich) treatment was used to eliminate the autofluorescence background, and sections were heated in a citrate buffer (pH 6.0) for 15 min to reverse formaldehyde crosslinks. After rinsing with PBS (pH 7.4), the sections were blocked with PBS containing 5% normal goat serum overnight at 4 °C. The sections were incubated with rabbit anti-*B. pseudomallei* or anti-*B. mallei* polyclonal antibody (1:1000–1500) for 2 h at room temperature. After rinsing with PBS, the sections were incubated with a secondary Alexa Fluor 488 conjugated goat anti-rabbit antibody for 1 h at room temperature. Sections were cover slipped using the Vectashield mounting medium with or without 4′,6-diamidino-2-phenylindole (DAPI) (Vector Laboratories, Burlingame, CA, USA) to stain nuclei. Additionally, in some cases, to visualize the presence of macrophages in the nonhuman primate samples, a mouse anti-human/NHP CD68 antibody was used (Dako Agilent Pathology Solutions) or CD45 antibody for lymphocytes (Dako). To visualize *B. pseudomallei/B. mallei*, z-stacks (multiple slices) were used. Images were captured on a Zeiss LSM 880 confocal system (Carl Zeiss, Oberkochen, Germany) and processed using ImageJ software (National Institutes of Health, Bethesda, MD, USA).

## 3. Results

### 3.1. IHC Analysis of FFPE by Bright Field Microscopy

**Figure 1:**
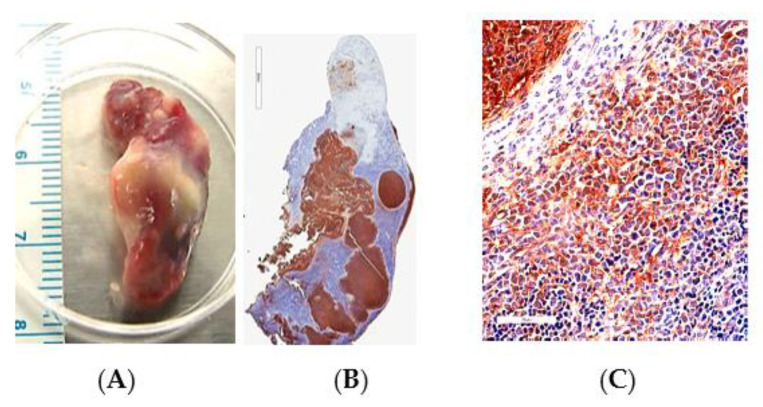
Detection of the exopolysaccharide of *B. pseudomallei* 22 in the spleen of a C57BL/6 mouse 47 days post-infection (PI) by bright field microscopy. (**A**). An enlarged spleen (1537 mg) with multifocal pyogranulomatous inflammation from the infected mouse. (**B**). IHC (low magnification) analysis revealed the presence of the exopolysaccharide (brownish color) from *B. pseudomallei 22* (scale bar, 4 mm). (**C**). IHC (high magnification) analysis of *B. pseudomallei* 22 infected spleen cells showed the stained exopolysaccharide material both within and adjacent to a positive stained pyogranuloma (scale bar, 70 µm).

In the animal model studies of melioidosis and glanders, bright field microscopy was used in the IHC analysis of FFPE tissue from exposed animals. An anti-*B. pseudomallei* K96243 polyclonal antibody was used as the primary antibody to detect the presence of *B. pseudomallei* in the FFPE tissue. An example is shown of a spleen from a C57BL/6 mouse 47 days post-infection (PI) that was exposed to *B. pseudomallei* 22 by aerosol (Figure 1). The exopolysaccharide from *B. pseudomallei* 22 was seen as a brownish staining material associated with pyogranulomatous lesions and the immediate surrounding cells (Figure 1C,D). However, the actual pathogen was not seen on closer examination.

### 3.2. LSCM Analysis of FFPE Tissue

**Figure 2:**
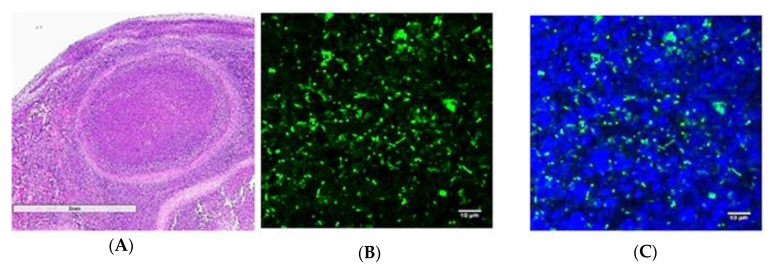
LSCM revealed the presence of *B. pseudomallei* 22 microorganisms in the spleen from a C57BL/6 mouse 47 days PI. (**A**). A H&E stained spleen section showing pyogranulomatous lesions in the spleen from Figure 1 (scale bar, 2 mm). (**B**). An anti-*B. mallei* GB18 antibody revealed the presence of *B. pseudomallei* (green) in the pyogranuloma (scale bar, 10 µm). (**C**). DAPI was used as a nuclear stain (blue) with the anti-*B. mallei* antibody to stain the nucleus of spleen cells in the pyogranuloma (scale bar 10 µm).

Because of the difference in technology between bright field microscopy and LSCM on how the image is captured, would this method enable us to see *B. pseudomallei* bacterial cells in FFPE tissues? A polyclonal antibody raised against a formalin-treated *B. mallei* GB18 was used as the primary antibody to examine FFPE tissue by LSCM from a C57BL/6 mouse exposed to *B. pseudomallei* 22. The same infected spleen shown in Figure 1 was used for comparison that was examined by bright field microscopy (see Table 1 for tissue source). Unlike the results with bright field microscopy, however, *B. pseudomallei* 22 cells were seen within the pyogranulomatous lesions in the spleen from the infected C57BL/6 mouse with LSCM (Figure 2). 

#### 3.2.1. LSCM Analysis of Murine Tissue from Animal Model Studies

**Figure 3:**
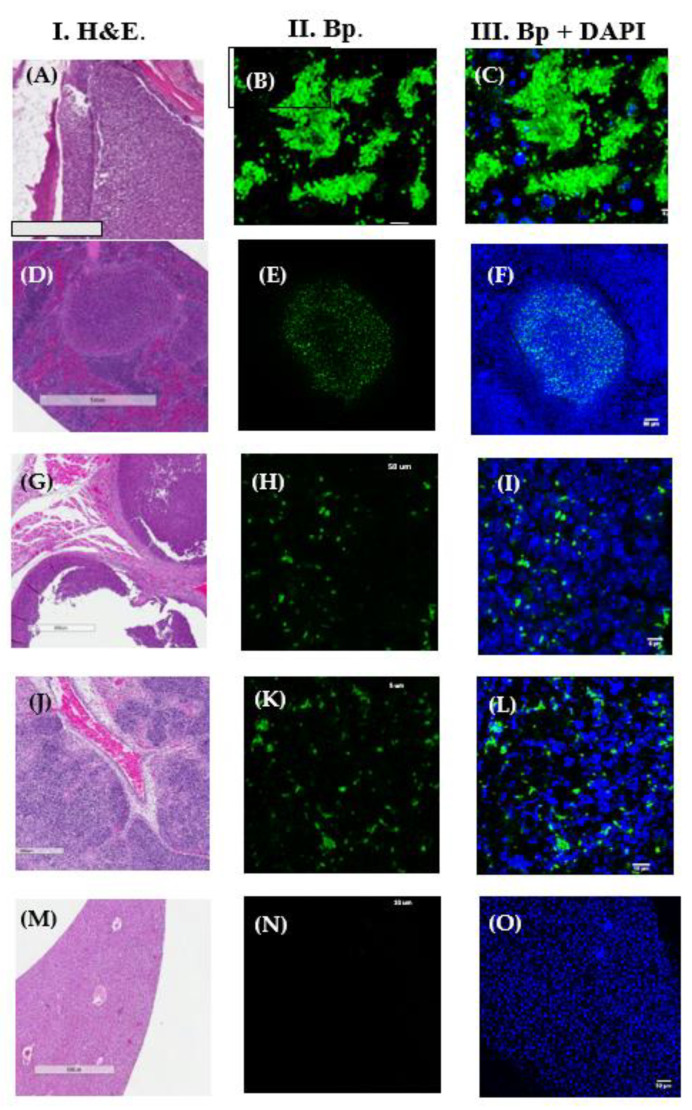
LSCM showed the presence of *B. pseudomallei* K96243 (BpK) in murine FFPE tissues. Ten mice (one to four organs in each mouse) were examined by LSCM. An anti-*B. mallei* GB18 antibody was used for the LSCM study. Column I shows the H&E stain of FFPE mouse tissue examined. Column II shows the presence of BpK (green) in tissue examined by LSCM, and column III shows BpK with the addition of DAPI to stain nuclei (blue) of tissue cells present. (**A**–**C**) show the dorsal thoracic region of a BALB/c mouse 22 days PI after aerosol exposure (scale bars, 900 µm, 5 µm, and 5 µm, respectively). (**D**–**F**) show the spleen section of a BALB/c mouse 22 days PI after aerosol exposure (scale bars, 1 mm, 50 µm, and 50 µm, respectively). (**G**–**I**) show the lumbar region of a BALB/c mouse 22 days after intraperitoneal injection of BpK (scale bars, 500 µm, 5 µm, and 5 µm, respectively). (**J**–**L**) show the lung of a BALB/c mouse 19 days PI after aerosol exposure (scale bars, 500, 10, and 10 µm, respectively). (**M**–**O**) show the liver of a C57BL/6 mouse that was negative (scale bars, 900, 50, and 50 µm, respectively).

**Figure A1:**
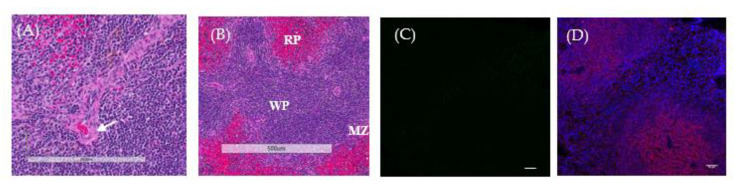
Examples of naïve BALB/c mouse spleens. (**A**) H&E of a mouse spleen showing possible vessel (arrow) entering the white pulp (scale bar 200 µm). (**B**) H&E of another naïve mouse spleen showing the white pulp (WP) and red pulp (RP) with the marginal zone (MZ) (scale bar 500 µm). (**C**) LSCM of BALB/c naïve spleen in A (scale bar 50 µm). (**D**) LSCM of spleen in A with DAPI (nuclei) and CD45 (lymphocytes) stains (scale bar 50 µm).

The previous results encouraged us to begin a retrospective study of archival FFPE tissues from our murine melioidosis animal model studies with LSCM to see if *B. pseudomallei* could be visualized in other animal tissues (see Table 1 for tissue source). FFPE tissues were examined from 10 mice (one to four organs from each mouse) that were exposed to *B. pseudomallei* by LSCM. Not all mice (2) were positive for *B*. *pseudomallei*. Tissues that were positive were also those with local pyogranulomatous inflammation, and areas without pyogranulomatous inflammation were negative for the pathogen (Figure 3). In Figure 3A–C, clusters of *B. pseudomallei* K96243 cells were seen in the dorsal thoracic region of a BALB/c mouse that showed staining of the outer surface of single and dividing cells of the microorganism. In pyogranulomas present in the spleen, the microorganism was seen primarily within the pyogranuloma, and very few were outside the pyogranuloma (Figure 3D–E). *B. pseudomallei* K96243 was seen in the lumbar (Figure 3G–I) and lung (Figure 3J–L) of aerosol exposed BALB/c mice. Figure 3M–O showed a negative liver from a C57BL/6 mouse that was exposed to *B. pseudomallei* K96243. See Figure A1 for LSCM analysis of spleens from naïve BALB/c mice.

#### 3.2.2. LSCM Analysis of Nonhuman Primate (NHP) Tissue

**Figure 4:**
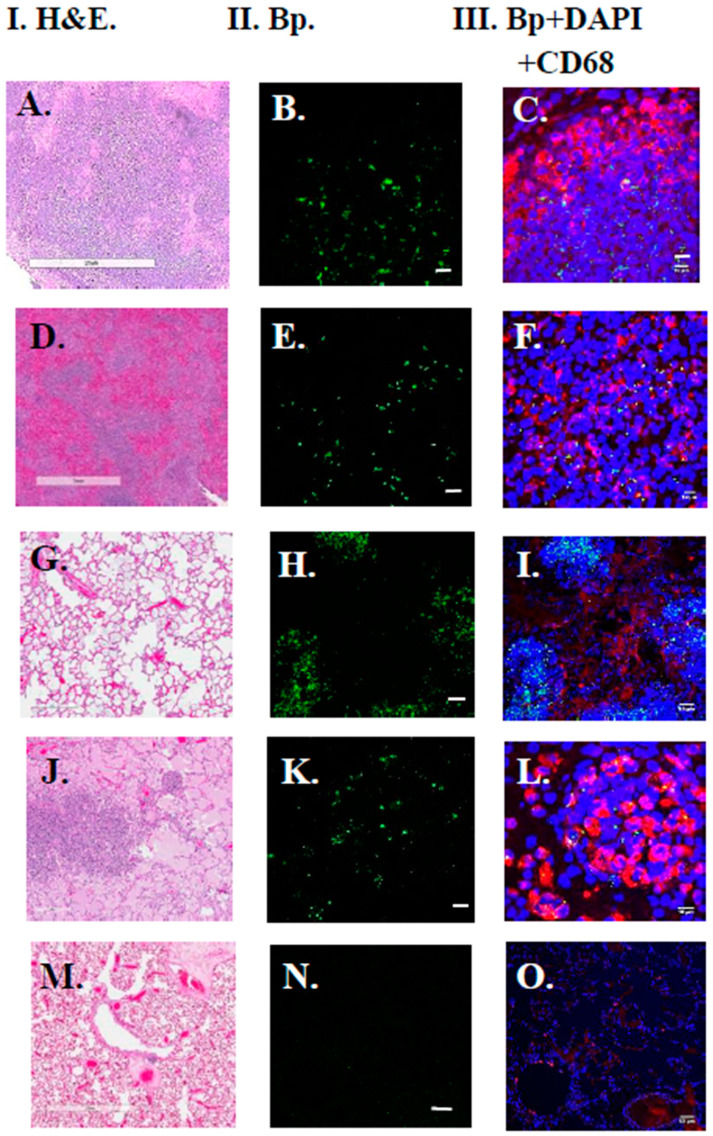
LSCM showed the presence of *B. pseudomallei* (Bp) HBPUB10134a in FFPE tissues of African Green monkeys (AGM) and Rhesus macaques. The liver, spleen, and lungs of two AGMs and two rhesus macaques were examined by LSCM after aerosol exposure to Bp HBPUB10134a. An anti-*B. mallei* GB18 antibody was used for the LSCM study. Column I shows the H&E stained sections examined from the nonhuman primates. Column II shows the presence of Bp (green) in the tissues examined by LSCM. Column III shows the presence of Bp with DAPI stained nuclei (blue) and anti-CD68+ antibody (macrophage, red) included. (**A**–**C**) show the presence of Bp HBPUB10134a in the lungs of an AGM 13 days PI (scale bars, 2 mm, 10 µm, and 10 µm, respectively). (**D**–**F**) show the presence of Bp HBPUB10134a in the spleen of an AGM 13 days PI (scale bars, 1 mm, 10 µm, and 10 µm, respectively). (**G**–**I**) show the presence of Bp HBPUB10134a in the lung of an AGM 5 days PI (scale bars, 400, 50, and 50 µm, respectively). (**J**–**L**) show the presence of Bp HBPUB10134a in the lung of a Rhesus macaque 13 days PI (scale bars, 300, 10, and 10 µm, respectively). (**M**–**O**) show the lungs of a Rhesus macaque 42 days PI that was negative (scale bars, 2 mm, 50 µm, and 50 µm, respectively).

FFPE tissues from four NHPs (two African Green Monkeys [AGM], and two Rhesus macaques) were examined by LSCM that were exposed to *B. pseudomallei* HBPUB10134a by aerosol. Three tissues were examined by LSCM (lung, liver, spleen) for each NHP (see Table 1 for tissue source). Both AGM were infected (all tissues examined), but only one rhesus appeared to be infected (all tissues examined). Generally, more *B. pseudomallei* HBPUB10134a microorganisms were found in AGM tissues when present than in tissues from Rhesus macaques. Figure 4 shows examples of NHP FFPE tissues examined by LSCM. Examples of *B. pseudomallei* HBPUB10134a in the lung (Figure 4A–C) and spleen (Figure 4D–F) of one of the AGMs is shown, and the pathogen was seen in the lung of the other AGM (Figure 4G–I). For one of the Rhesus macaques, the microorganism was seen in the lung (Figure 4J–L), while in the other Rhesus macaque, no *B. pseudomallei* HBPUB10134a was present in the lungs (Figure 4M–O) or in other tissues examined. Although macrophages (CD68+) in the area of the infection or pyogranuloma were seen, it was difficult to ascertain if any of the B. pseudomallei HBPUB10134a were within the macrophages.

#### 3.2.3. LSCM Analysis of Suspected Human Tissue

**Figure 5:**
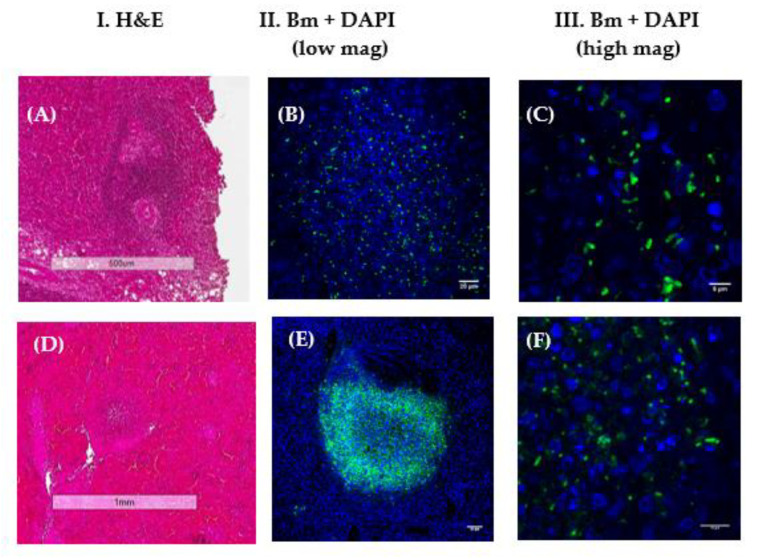
LSCM examination of a spleen biopsy from a human suspected of exposure to *B. mallei*. Column I shows the H&E stained areas of pyogranulomatous inflammation examined. Column II shows the results of the LSCM analysis of the same region containing positive cells (Bm, green) with DAPI (blue) at lower magnification. Column III shows the LSCM results (Bm, green) with DAPI (blue) stained nuclei at higher magnification. An anti-*B. mallei* GB18 antibody (1/1500 dilution) was used as the primary probe. (**A**–**C**) show the possible presence of *B. mallei* in one area of pyogranulomatous inflammation (scale bars, 500, 20, and 5 µm, respectively). (**D**–**F**) show the possible presence of *B. mallei* in another area with a relatively small pyogranuloma (scale bars, 1 mm, 50 µm, and 20 µm, respectively).

Other FFPE tissues were examined to determine if LSCM could identify the presence of *B. mallei*. A historical spleen biopsy (FFPE) from a human suspected of having been exposed to *B. mallei* was obtained. Figure 5 shows examples of two areas of pyogranulomatous inflammation in the spleen biopsy. In Figure 5A–C, positive cells were seen within an area of pyogranulomatous inflammation by LSCM that upon closer examination looked like bacterial rods (Figure 5C). In another area with a small pyogranuloma (Figure 5D–F), a cluster of positive cells (Figure 5E) was seen that appeared to consist of rod-shaped bacterial cells at higher magnification (Figure 5F). Thus, the possible presence of *B. mallei* in FFPE tissue was demonstrated from a human suspected of being exposed to *B. mallei* by LSCM. 

#### 3.2.4. Comparison of Polyclonal Antibodies Used to Examine FFPE Tissue by LSCM

**Figure 6:**
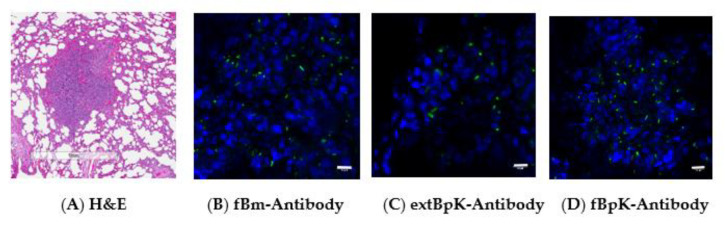
Different antibody preparations can be used with LSCM to identify *B. mallei* in FFPE tissue. All antibody preparations were diluted 1/1000 before use. Antibody preparations were tested at least twice on different tissues by LSCM. (**A**). H&E stained section of a lung from an AGM exposed to *B. mallei* FMH showed an area of pyogranulomatous inflammation (scale bar 1 mm). (**B**). LSCM used with an antibody raised against formalin-treated *B. mallei* GB18 (fBm) with DAPI stain (blue nuclei) showed the presence of positive cells (green) (scale bar 10 µm). (**C**). LSCM used with an antibody raised against an extract of *B. pseudomallei* K96243 (extBpK) cells with DAPI stain (blue nuclei) showed the presence of positive cells (green) (scale bar 10 µm). (**D**). LSCM used with an antibody raised against a formalin-treated, whole cells of *B. pseudomallei* K96243 (fBpK) with DAPI stain (blue nuclei) showed the presence of positive cells (green) (scale bar 10 µm).

A question arose if other types of antibodies would work with LSCM when examining FFPE tissue. In the previous study above with LSCM, an antibody was used that was developed against a formalin-treated *B. mallei* GB18 (fBm) whole cell. Figure 6A shows an area with pyogranulomatous inflammation in the lung from an AGM exposed to *B. mallei* FMH that was examined with two other antibody preparations (see Table A1 for antibody ELISA titers). Figure 6B shows the presence of *B. mallei* FMH in the area of inflammation with the antibody (raised against fBm) that was used in the previous study. In Figure 6C, similar results were seen with an antibody raised against a whole-cell extract of *B. pseudomallei* K96243 (extBpK). Similarly, the presence of *B. mallei* was seen in the lung of the AGM with an antibody raised against formalin-treated *B. pseudomallei* K96243 whole-cells (fBpK). Therefore, it appears that antibodies raised against three different *Burkholderia* antigen preparations will work with LSCM of infected FFPE tissue.

## 4. Discussion

The presence of *B. pseudomallei* or *B. mallei* bacterial cells in FFPE tissue by LSCM was demonstrated that it was not by bright field microscopy. *Burkholderia* cells in FFPE tissues were seen from mice and nonhuman primates exposed to *B. pseudomallei* or *B. mallei*, and in a historical spleen biopsy from a human suspected of being exposed to *B. mallei*. The difference in the technology of image formation between bright field microscopy and LSCM made it possible to clearly visualize the pathogen in FFPE tissue. Briefly, in LSCM the excitation (laser) and emission light sources are limited (either both or only emission) by pinhole apertures with the focus on a point(s) within a single plane of the sample. Excitation and emission light derived from above, below, and away from the point or plane of focus are generally excluded by the pinhole apertures that result in a higher resolution image. As the image is scanned, the point of focus stays in the same plane (optical sectioning) [14]. In contrast, in bright field microscopy, the entire field is exposed by the light source, and the resulting image may focus on multiple planes which results in a lower resolution image than obtained with LSCM. This resolution is also true with epi-fluorescence microscopy when compared with LSCM.

In earlier reports on the visualization of *B. pseudomallei* or *B. mallei* in FFPE tissue samples, it was reported that the presence of the pathogen was seen after immunohistochemical (IHC) staining of paraffin sections [15,16], which was different than in our present report because the pathogen after IHC staining was not visualized. It was not clear why there was a difference from our study, but in Wong et al. [15], they reported using a Gram stain on their FFPE tissues, while in Glaros et al. [16], they may have used a different imaging system. In other reports, fluorescent in situ hybridization (FISH) was used to identify *B. pseudomallei* or *B. mallei* present in FFPE tissue in infected murine [17] or human tissue samples [18]. Furthermore, DNA was extracted from infected murine FFPE tissue samples and polymerase chain reaction (PCR) was run with *B. pseudomallei* specific primers to determine if the pathogen was present in the samples [19,20]. In two other reports, transmission electron microscopy was used to visualize *B. pseudomallei* in experimental mouse studies [21] or clinical human melioidosis patients [22] without or with FFPE tissue, respectively. 

There are few reports of the use of LSCM to visualize *B. pseudomallei* or *B. mallei* in infected tissue. LSCM was used to visualize *B. mallei*, *B. pseudomallei*, or *B. thailandensis* in murine macrophage-like RAW 264.7 cells [23,24], and *B. pseudomallei* infection of A549 human lung epithelial cells [22,25]. A recent study of *B. mallei* in an infected mouse FFPE spleen sample that was examined by LSCM was reported [26]. Finally, LSCM has been used to detect the presence of other pathogens, such as *Mycobacterium tuberculosis* in lung tissue from human patients [27]. 

One caution to this report is that the figures presented in this report may show more microorganisms than present than seen in human cases of melioidosis or glanders. Animal tissues are easier to recover and manipulate than human tissue, for example the mouse spleen. *Burkholderia* appear to accumulate in the spleen and cause the formation of pyogranulomas that are in most cases easy to observe upon autopsy. A mouse spleen with a pyogranuloma may contain from 10^4^ to 10^9^ CFU [9]. Additionally, because these tissues come from experimental melioidosis or glanders animal models, they may be exposed to more CFU than normally encountered by humans that acquired melioidosis or glanders. Thus, they are more likely to be acutely infected. In addition, animals may be chosen that are more susceptible to infection than others, such as BALB/c mice versus C57BL/6 mice, where the latter species is generally more resistant than the former. This could result in higher CFU in the susceptible animal than in the resistant animal [7,8].

This report established the feasibility of using LSCM to validate the presence of *B. pseudomallei* or *B. mallei* in FFPE tissue from different animal or human sources. This technology may be diagnostic for melioidosis (or glanders) or complement the diagnosis of the disease with the isolation of the pathogen. In addition, it would be useful to have *B. pseudomallei* and *B. mallei* capsule-specific monoclonal antibodies, if there is a question between diagnosis of melioidosis or glanders [28,29,30]. One advantage of LSCM is that it can validate the diagnosis of melioidosis or glanders, but some disadvantages of LSCM are the cost of the system, the expertise to operate the system, and the preparation of the sample would limit its usefulness in an on-site clinical setting where diagnosis is needed. At present, it may have to be part of a core facility that serves a wide area in need of such supporting technology.

## Figures and Tables

**Table 1:** Summary of tissues from mice, nonhuman primates, or human evaluated by LSCM.

Figure No.	Strain/Species ^a^	Exposure to Bp or Bm Strain	Amount of Exposure (CFU) ^b^	Route of Infection	Tissue	Time Post-Infection (Days)	CFU/g Tissue	Source of Tissue
2	C57BL/6	Bp 22	127	Aerosol	Spleen	47	na ^d^	This ref
3	BALB/c	Bp K96243	3.0 × 10^4^	IP^c^	Dorsalthoracic	22	na	[7]
3	BALB/c	Bp K96243	5.0	Aerosol	Spleen	15	na	[7]
3	BALB/c	Bp K96243	3.0 × 10^4^	IP	Lumbar	22	na	[7]
3	BALB/c	Bp K96243	5.0	Aerosol	Lung	19	na	[7]
3	C57BL/6	Bp K96243	18.0	Aerosol	Liver	28	na	[7]
4	AGM	Bp HBPUB10134a	319	Nose only	Lung	13	158,489	This Ref
4	AGM	Bp HBPUB10134a	319	Nose only	Spleen	13	5,011,872	This Ref
4	AGM	Bp HBPUB10134a	420	Nose only	Lung	5	1,584,893	This Ref
4	Rhesus	Bp HBPUB10134a	286	Nose only	Lung	13	15,848,931	This Ref
4	Rhesus	Bp HBPUB10134a	531	Nose only	Lung	42	0	This Ref
5	Human	*B. mallei*	Unknown	Unknown	Spleen	Unknown	Unknown	Unknown
6	AGM	*B. mallei*	2.10 × 10^7^	Head only	Lung	14	Unknown	This Ref

^a^ AGM, African Green Monkey; Rhesus, Rhesus macaque. ^b^ Unknown: Do not have the information. ^c^ IP, intraperitoneal. ^d^ na, not applicable (or unknown). The whole tissue was fixed for immunohistochemical analysis.

## Appendix A

**Table A1:** Antibody titers of rabbit anti-*B. pseudomallei* and anti-*B. mallei* sera.

	ELISA Titer ^a^		
Antigen Used for Antibody Production	Type Antigen ^b^		
	IRBpK	fBpK	IRBmFMH
A.			
None (naïve rabbit serum)	6400 (1.00)	126 (1.26)	1008 (1.26)
BpK whole-cell extract (extBpK)	507,968 (1.26)	806,349 (1.26)	6,400,000 (1.00)
Formalin-treated Bm GB18 (fBm)	100,794 (1.26)	320,000 (1.00)	1,015,937 (1.26)
B.			
None (naïve rabbit serum)	5080 (1.26)	159 (1.26)	2016 (1.26)
Formalin-treated BpK (fBpK) (no. 1) ^c^	1,600,000 (1.00)	6,400,000 (1.00)	2,560,000 (1.00)
Formalin-treated BpK (fBpK) (no. 2)	1,600,000 (1.00)	3,200,000 (1.00)	4,063,747 (1.26)

^a^ Antibody titers were determined at least twice as previously described [12]. ^b^ Antigens used in ELISA: irradiated BpK96243 (IRBpK); formalin BpK96243 (fBpK); irradiated Bm FMH (IRBmFMH). ^c^ Antibody used for Figure 6D.
